# Expression of CXCR4 and breast cancer prognosis: a systematic review and meta-analysis

**DOI:** 10.1186/1471-2407-14-49

**Published:** 2014-01-29

**Authors:** Zhigang Zhang, Chao Ni, Wuzhen Chen, Ping Wu, Zhen Wang, Junhua Yin, Jian Huang, Fuming Qiu

**Affiliations:** 1 Cancer Institute (Key Laboratory of Cancer Prevention & Intervention, National Ministry of Education, Provincial Key Laboratory of Molecular Biology in Medical Sciences), The Second Affiliated Hospital, Zhejiang University School of Medicine, Hangzhou 310009, China; 2 Department of Oncology, Second Affiliated Hospital, Zhejiang University School of Medicine, Hangzhou 310009, China

**Keywords:** Breast cancer, CXCR4, Prognosis

## Abstract

**Background:**

The chemokine receptor CXCR4 plays a significant role in biological processes, as well as in tumorigenesis and the progression of cancer, especially breast cancer. However, the clinical application of CXCR4 for breast cancer prognosis is still very limited. A meta-analysis based on published studies was performed with the aim of obtaining an accurate evaluation of the relationship between CXCR4 expression and the prognosis of breast cancer.

**Methods:**

A comprehensive search strategy was used to search relevant literature in PubMed, MEDLINE and the ISI Web of Science. The correlation between CXCR4 expression and clinicopathological features and breast cancer prognosis was analyzed. This meta-analysis was carried out using Review Manager 4.2.

**Result:**

Thirteen eligible studies consisting of 3865 participants were included. We found that breast cancers with CXCR4 expression were associated with lymph node status (pooled RR =1.20, 95% CI: 1.01-1.43, P<0.001) and distant metastasis (pooled RR =1.52, 95% CI: 1.17-1.98, P = 0.125). CXCR4 overexpression was significantly associated with disease free survival (DFS) (RR = 0.77, 95% CI = 0.70–0.86, P = 0.554) and overall survival (OS) (RR = 0.70, 95% CI = 0.59–0.83, P = 0.329). However, there was no significant association between CXCR4 expression and some clinical parameters of breast cancer, such as tumor category, ER status, PR status, or c-erbB-2 status.

**Conclusion:**

Our meta-analysis showed that CXCR4 is an efficient prognostic factor for breast cancer. Overexpression of CXCR4 was significantly associated with lymph node status and distant metastasis and indicated poor overall and disease free survival.

## Background

Breast cancer is the most common form of cancer diagnosed in women. Thus far in 2013, breast cancer has accounted for 29% of all new cancer cases and 14% of all cancer deaths among women worldwide [1]. Breast cancer-related mortality is associated with the development of metastatic potential of the primary tumor. Recently, many studies have shown that the presence of CXCR4 can signify invasion and metastasis in several cancers, including breast cancer [2].

The chemokine receptor CXCR4 is a 352-amino acid rhodopsin-like G protein-coupled receptor (GPCR) that selectively binds the CXC chemokine stromal cell-derived factor 1 (SDF-1), also known as CXCL12. This chemokine receptor has been identified to play a crucial role in a number of biological processes, such as trafficking and homeostasis of immune cells such as T lymphocytes [3], and the CXCL12/CXCR4 axis is known to be important in the enhancement of stem cell homing in tissue regeneration [4]. In various types of cancer, CXCR4 plays a vital role in tumorigenesis and the progression of cancer [5,6]. A potential mechanism of CXCR4’s involvement in tumor dissemination and metastasis is through promoting its transendothelial migration at the primary site [7]. Further evidence indicates that CXCR4 not only affects breast cancer metastasis but also promotes the survival and proliferation of breast cancer cells through increasing the number of blood vessels in tumors [8]. However, there are insufficient studies to confirm the clinical significance of CXCR4 in breast cancer, and its accurate prognostic value in breast cancer is still unclear, especially in the different molecular types of breast cancer. To address this issue, we conducted a meta-analysis aimed at evaluating the value of CXCR4 as a prognostic marker for breast cancer and to determine the relationship between CXCR4 and several clinicopathological features of breast cancer.

## Methods

### Publication search

This systematic review and meta-analysis is reported according to the Preferred Reporting Items for Systematic Reviews and Meta-Analyses (PRISMA) statement [9]. The electronic databases PubMed (http://www.ncbi.nlm.nih.gov/pubmed/), MEDLINE and ISI Web of Science were searched using the following tags: “CXCR4” and “breast cancer”. The citation lists associated with the studies, including review articles, that were retrieved in the search were used to identify additional relevant publications. The articles utilized in this study were published up to March 2013. The title and abstract of each study identified in the search were scanned to exclude any clearly irrelevant reports.

### Selection criteria

The studies included in this meta-analysis were either randomized controlled studies (RCTs) or observational studies (case–control or cohort) that evaluated the association between CXCR4 expression and breast cancer. The criteria for inclusion were as follows: a) articles evaluating the relationship between CXCR4 expression and parameters such as clinicopathological features and prognostic factors of breast cancer; b) articles containing sufficient published data to determine an estimate of relative risk (RR) and a 95% confidence interval (95% CI); and c) full text, original research articles published in English.

Letters to the editor, reviews, comments, duplicated studies and articles published in books as well as papers published in non-English languages were excluded.

### Data extraction

All data were independently abstracted by two reviewers with standardized data abstraction tools. Disagreements in data extraction were resolved by consensus and by referring back to the original article. The following data were obtained from each article: first author’s last name; year of publication; country of the population studied; number of participants; duration of follow-up; staining methods of CXCR4; staining patterns of CXCR4; the choice of cutoff scores for the definition of positive staining or staining intensity; T category (T0-2, T3-4); N category; distant metastasis; c-erbB-2, ER and PR status; and most importantly, the 5-year overall survival (OS) and disease-free survival (DFS) rates.

Because the cutoff value for the CXCR4 group varied among studies, we defined CXCR4-high expression values according to the original articles. The T category was determined according to the American Joint Committee on Cancer (AJCC) cancer staging manual (one group: T0-2, other group: T3-4). To avoid bias from studies contributing very long-term follow-up data compared with other studies, both OS and DFS were standardized to include 5 years of follow-up in all studies. For the articles that did not provide 5-year OS and DFS rates directly, Kaplan–Meier curves were evaluated using GetData Graph Digitizer 2.24 (http://getdatagraph-digitizer.com).

### Statistical analysis

Statistical analyses were performed according to the guidelines proposed by the Meta-Analysis of Observational Studies in Epidemiology group. The relative risk (RR) with 95% confidence interval (95% CI) was calculated using Review Manager 4.2. Study heterogeneity was measured using the Q test and I^2^ test. Fixed-effects models (Mantel-Haenszel, P>0.1 and I^2^<50%) assume that the differences between the results of various studies are due to chance. Random-effects models (DerSimonian and Laird, P ≤ 0.1 or I^2^ ≥ 50%) assume that the results could genuinely differ between studies. In the absence of heterogeneity, both fixed- and random-effects models provide similar results. When heterogeneity is present, the random-effects model is considered to be more appropriate than a fixed-effects model, resulting in wider intervals and a more conservative estimate of treatment effect. The potential for publication bias was assessed using the Begg rank correlation method and the Egger weighted regression method (software Stata 11.0, P<0.05 was considered representative of statistically significant publication bias). All P values are two tailed.

## Results

### Search results

**Figure 1:**
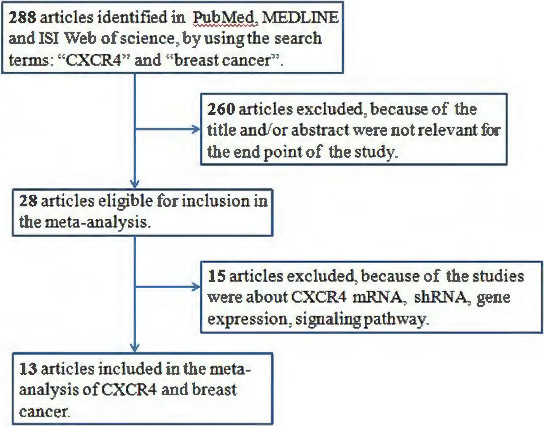
Flowchart of the selection of studies for inclusion in the meta-analysis.

The detailed search steps are described in Figure 1. Initially, 288 articles were retrieved utilizing the search strategy described above. After titles and abstracts were reviewed, 260 articles were excluded due to the end point of the study; some studies did not provide data between CXCR4 expression and pathological features or specify whether disease free survival (DFS)/overall survival (OS) was investigated. Of the published articles, 15 studies were excluded because they were about CXCR4 mRNA, CXCR4 shRNA, CXCR4 gene expression or the CXCR4 signaling pathway. Finally, a total of 13 studies were included in the meta-analysis.

### Characteristics of eligible studies

All features of the 13 eligible studies are listed in Table 1. These thirteen observational retrospective studies, consisting of approximately 3865 participants with a median of 297 (from 54 to 1699) per study, were included. These studies evaluated the expression of CXCR4 and evaluation parameters for breast cancer, based on either clinicopathological features or prognostic factors. Among them, 7 were from the USA, 1 was from Canada, 3 were from China, 1 was from Japan, and 1 was from Korea. We did not obtain any studies from Europe. In addition, the expression of CXCR4 was detected by immunohistochemistry (IHC) in 3 studies, by tissue microarray (TMA) in 2 studies and by western blot analysis in 6 studies.

**Table 1:** Characteristics of the included studies

**Author**	**Year**	**Country**	**Number of patients**	**Duration of follow-up**	**Methods**	**Staining patterns**	**Cut off scores (high/low)**	**T category (T0/T1/2/3/4)**	**N category (P/N)**	**Distant metastasis (M1/M0)**	**State of CerbB-2 (P/N)**	**Stata of ER (P/N)**	**Stata of PR (P/N)**	**5-year OS rate**	**5-year DFS rate**
Zhang [10]	2012	China	232	NA	IHC	Membrane or cytoplasm	5% (134/98)	H:(0/48/86/0/0); L:(0/38/60/0/0)	H:(70/64); L:(28/70)	NA	H:(82/52); L:(38/60)	H:(52/82); L:(44/54)	H:(44/90); L:(38/60)		
PARKER [11]	2012	USA	185	54 months	Western blots	Membrane and cytoplasm	≥7.5 F (37/148)	H:(0/7/21/7/2); L:(2/21/82/24/18)	H:(37/37); L:(148/148)	NA	H:(14/20); L:(39/91)	H:(12/23); L:(51/97)	H:(11/24); L:(45/103)	H:57% (21/37) L:69% (102/148)	H:53% (20/37) L:62% (92/148)
Hiller [12]	2011	USA	77	42 months	Western blots	Membrane and cytoplasm	≥6.6 F (22/55)	LABC(NA)	NA	NA				H:50% (11/22) L:78% (43/55)	H:41% (9/22) L:67% (37/55)
Chu [13]	2010	USA	101	59 months	Western blots	Membrane and cytoplasm	≥6.6 F (22/79)	H:(0/8/11/3/0); L:(0/58/18/3/0)	NA	NA				H:81% (18/22) L:97% (77/79)	H:79% (17/22) L:96% (76/79)
Liu [14]	2010	China	200	88 months	TMA	Cytoplasm	Score ≥ 6 (110/90)	H:(0/41/56/13/0); L:(0/27/50/13/0)	H:(69/41); L:(31/59)	NA	H:(32/78); L:(15/75)	H:(59/51); L:(41/49)	H:(60/50); L:(39/51)	H:68% (75/110) L:80% (72/90)	
Liu* [14]	2010	China	200	88 months	TMA	Nuclear	Score ≥ 6 (113/87)	H:(0/32/67/14/0); L:(0/36/39/12/0)	H:(61/52); L:(39/48)	NA	H:(87/26); L:(21/66)	H:(62/51); L:(38/49)	H:(62/51); L:(37/50)	H:63% (71/113) L:82% (71/87)	
Mizell [15]	2009	USA	115	53 months	Western blots	Membrane and cytoplasm	≥6.6 F (13/102)	H:(0/2/10/0/1); L:(0/41/50/10/1)	H:(6/7); L:(31/71)	H:(5/8); L:(14/88)	H:(0/13); L:(0/102)	H:(2/11); L:(37/65)	H:(2/11); L:(34/68)	H:52% (7/13) L:86% (88/102)	H:38% (5/13); L:74% (75/102)
ANDRE [16]	2009	USA	794	10 years	IHC	Membrane or cytoplasm	1% (92/702)	H:≥3 (9/81); L: ≥3 (61/637)	H:(40/51); L:(300/402)	H:(28/64); L:(155/547)	H:(10/79); L:(88/607)	H:(41/36); L:(378/238)			
Yasuoka [17]	2008	Japan	113	5 years	IHC	Cytoplasmic	Score ≥ 5 (56/57)	T1:H:15; L: 19 T2-4:H:41; L: 28	H:(36/20); L:(23/34)	H:(22/34); L:(7/50)	H:(19/39); L:(15/42)	H:(32/24); L:(36/21)	H:(29/27); L:(33/24)		
Yasuoka* [17]	2008	Japan	113	5 years	IHC	Nuclear	Score ≥ 5 (29/84)	T1:H:11; L: 23 T2-4:H:18; L: 61	H:(13/16); L:(46/38)	H:(7/22); L:(22/62)	H:(7/22); L:(25/59)	H:(18/11); L:(50/34)	H:(17/12); L:(45/39)		
Woo [18]	2007	Korea	107	NA	IHC	Cytoplasm	Score ≥ 4 (33/72)	H:(0/7/18/8/0); L:(0/19/45/8/0)	H:(21/12); L:(34/38)		H:(17/15); L:(30/34)	H:(20/13); L:(41/27)	H:(20/13); L:(38/28)		
Woo* [18]	2007	Korea	107	NA	IHC	Nuclear	Score ≥ 4 (63/42)	H:(0/16/37/10/0); L:(0/10/26/6/0)	H:(39/24); L:(16/26)		H:(31/28); L:(16/21)	H:(39/23); L:(22/17)	H:(36/24); L:(22/17)		
Holm [19]	2007	USA	103	26 months	Western blots	Membrane and cytoplasm	≥6.6 F (41/62)	H:(0/7/27/5/2); L:(0/23/32/7/0)	H:(23/17); L:(33/30)						H:46% (19/41); L:70% (43/62)
Su [20]	2006	Taiwan	85	NA	IHC	Cytoplasm	Score ≥ 3 (10/75)	H:(0/2/8/0/0); L:(0/34/41/0/0)	H:(5/2); L:(21/41)	H:(1/9); L:(8/67)	H:(6/3); L:(38/25)	H:(7/3); L:(44/24)	H:(3/7); L:(41/27)		
Su* [20]	2006	Taiwan	85	NA	IHC	Nuclear	Score ≥ 3 (59/26)	H:(0/27/32/0/0); L:(0/9/17/0/0)	H:(15/30); L:(11/13)	H:(6/53); L:(3/23)	H:(30/19); L:(14/9)	H:(33/20); L:(18/7)	H:(29/24); L:(15/10)		
Salvucci [21]	2006	Canada	1699	68 months	TMA	Cytoplasm	>50%, 30–50, <30 (121/590/986)		H:(40/66); M:(112/242); L:(422/460)		H:(108/705); M:(90/390) L:(32/57)	H:(63/58); M:(436/154) L:(807/179)	H:(19/102); M:(180/396); L:(359/597)		
Salvucci* [21]	2006	Canada	1697	69 months	TMA	Nuclear	30% (1161/538)		H:(514/527); L:(218/242)		H:(160/809); L:(70/343)	H:(895/266); L:(412/126)	H:(390/743); L:(166/353)		
Holm [22]	2009	USA	54	30 months	Western blots	Membrane and cytoplasm	≥6.6 F (19/35)							H:15% (3/19) L:40% (14/35)	H:20% (4/19); L:55% (19/35)

*Date from the same study of the previous line.

### Correlation of CXCR4 expression with clinicopathological parameters

**Figure 2:**
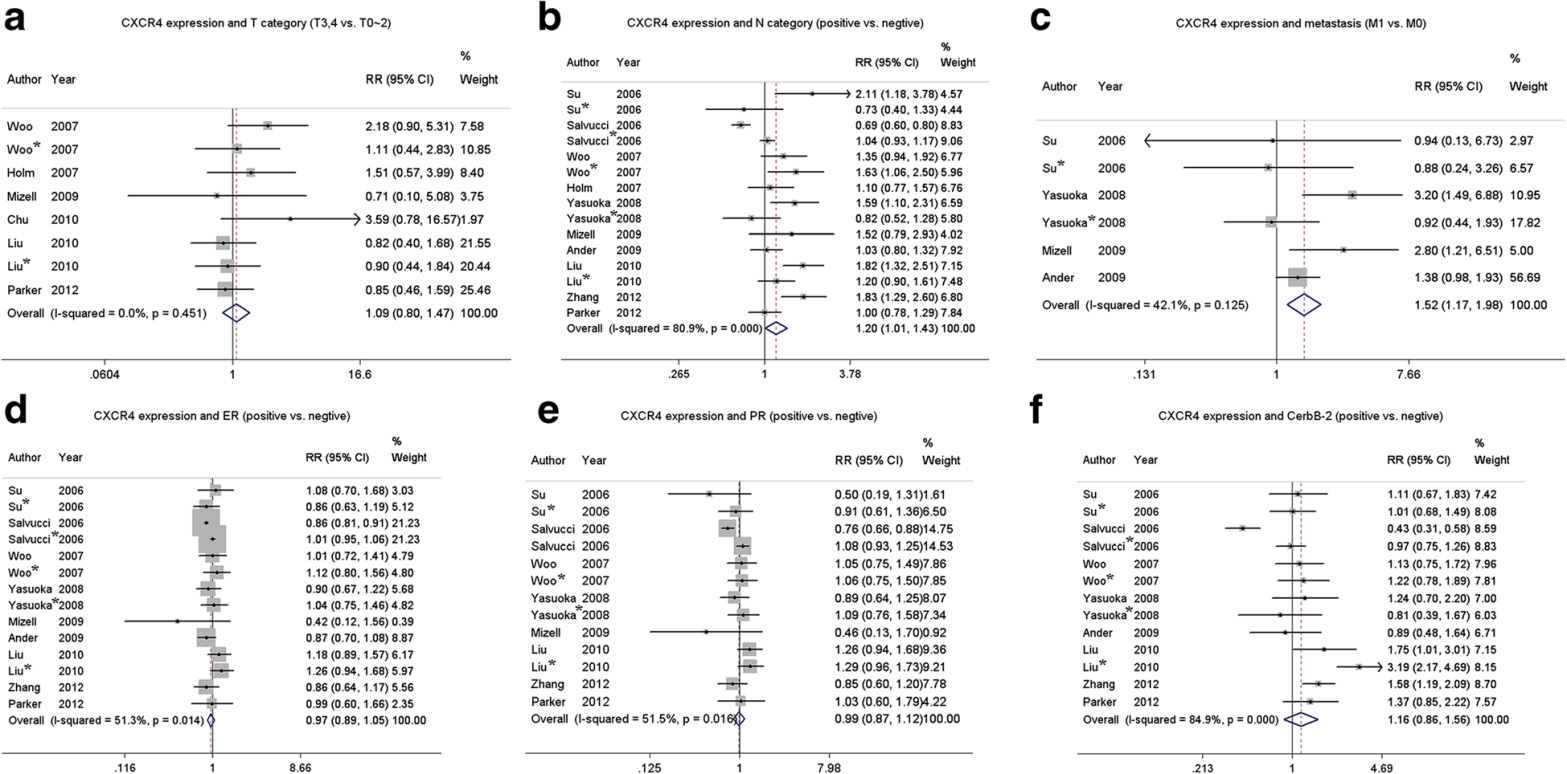
The forest plot of RRs was assessed for association between CXCR4 and clinicopathological features such as tumor category (a), N category (b), distant metastasis (c), ER status (d), PR status (e) and c-erbB-2 status (f).

CXCR4 expression was not associated with certain clinical parameters of breast cancer, such as tumor category (T category: T0-2, T3-4) (pooled RR =1.09, 95% CI: 0.80-1.47, P = 0.451 and I^2^ = 0 fixed-effect), ER status (pooled RR =0.97, 95% CI: 0.97-1.05, P = 0.014 and I^2^ = 51.3 random-effect), PR status (pooled RR =0.99, 95% CI: 0.87-1.12, P = 0.016 and I^2^ = 51.5 random-effect), or c-erbB-2 status (pooled RR =1.16, 95% CI: 0.85-1.56, P<0.001 and I^2^ = 84.9 random-effect) (Figure 2a,d,e,f).

However, breast cancers with CXCR4 expression were associated with lymph node status (pooled RR =1.20, 95% CI: 1.01-1.43, P<0.001 and I^2^ = 80.9 random-effect) and distant metastasis (pooled RR =1.52, 95% CI: 1.17-1.98, P = 0.125 and I^2^ = 42.1 random-effect) (Figure 2b,c). In the staining pattern subgroup analysis, CXCR4 membrane and/or cytoplasm expression (pooled RR =1.72, 95% CI: 1.29-2.29, P = 0.121 and I^2^ = 48.4 random-effect) had a more significant association with distant metastasis than nuclear CXCR4 expression. Additional results from the subgroup analyses can be found in Additional file 1: Tables S1 and Additional file 2: Tables S2.

### Impact of CXCR4 expression on 5-year OS and DFS rates

**Figure 3:**
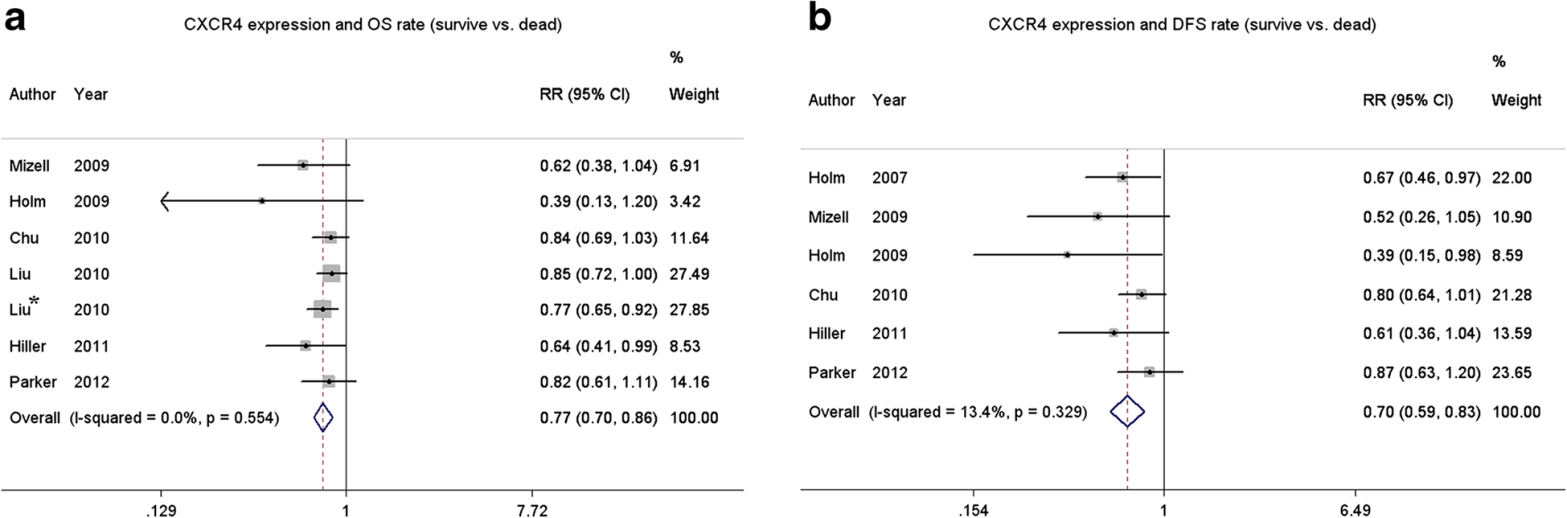
The forest plot of RRs for the 5-year OS rate (a) and 5-year DSF rate (b) among the included studies.

The relationship between CXCR4 expression and breast cancer prognosis is illustrated in Figure 3. Six studies (including a total of 732 patients) that demonstrated the association of CXCR4 and the 5-year OS rate were obtained from the published information, and six studies (including a total of 635 patients) containing information on the correlation between CXCR4 and the 5-year DFS rate were obtained from the published articles. CXCR4 overexpression was statistically associated with a poor 5-year OS rate (Figure 3a, RR = 0.77, 95% CI = 0.70–0.86, P = 0.554 and I^2^ = 0 fixed-effect). The 5-year OS rate was 0.77-fold lower in CXCR4-positive patients. Furthermore, a similar difference was also observed in the 5-year DFS rate (Figure 3b, RR = 0.70, 95% CI = 0.59–0.83, P = 0.329 and I^2^ = 13.4 fixed-effect).

### Publication bias

**Figure 4:**
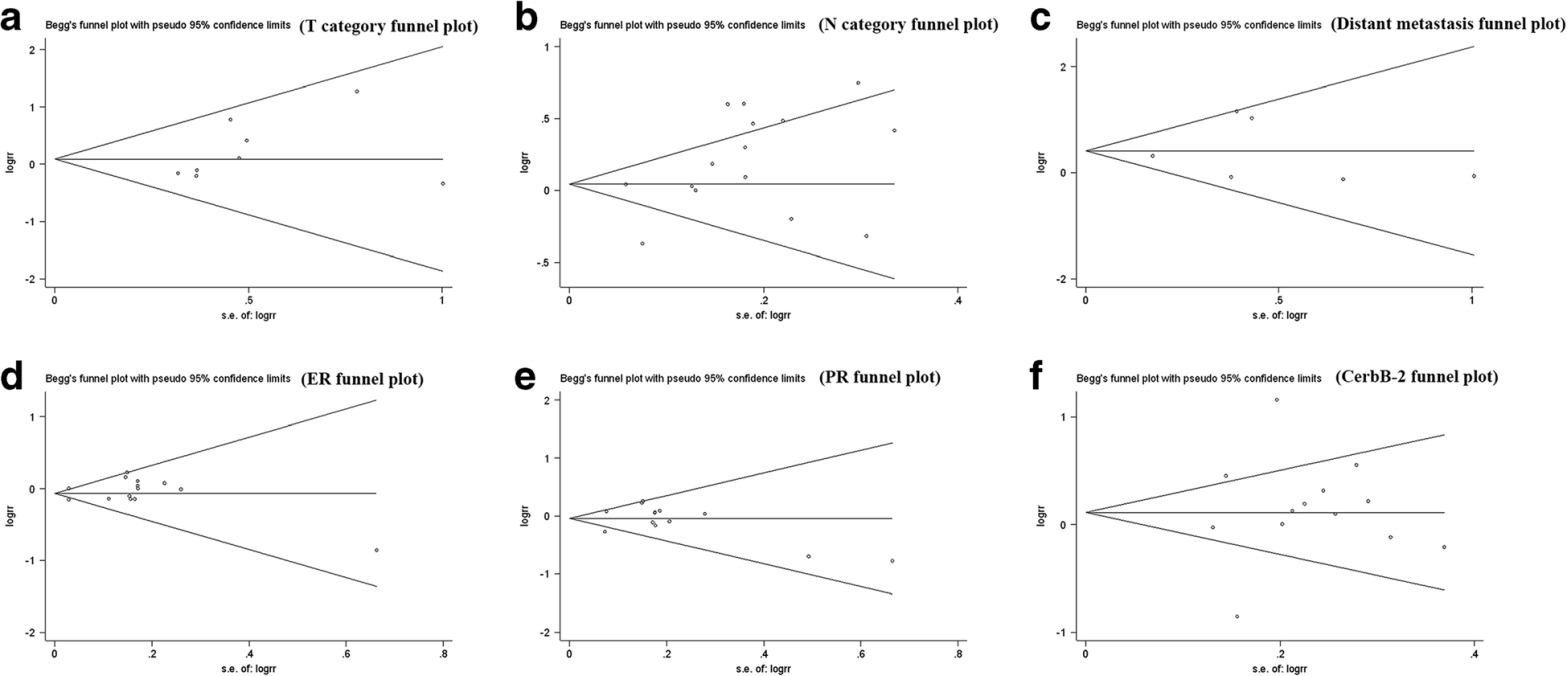
Begg’s test results of CXCR4 and clinicopathological features such as tumor category (a), N category (b), distant metastasis (c), ER status (d), PR status (e) and c-erbB-2 status (f).

**Figure 5:**
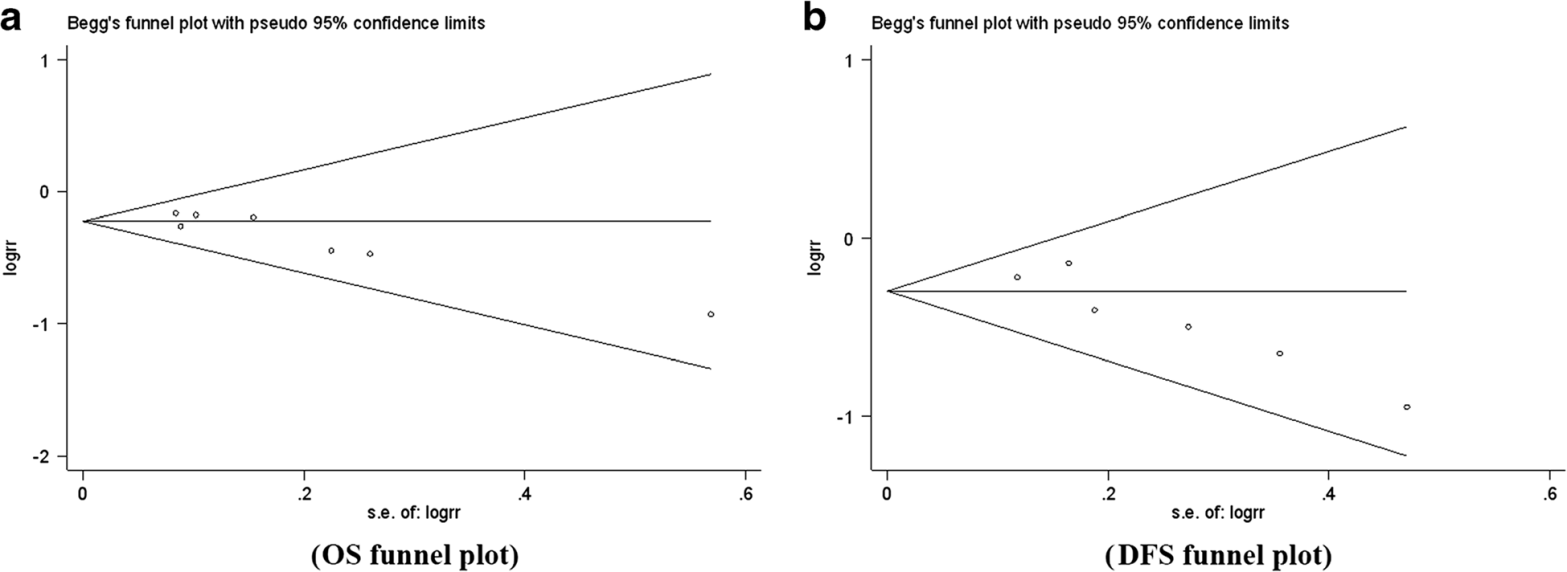
Begg’s test results of the 5-year OS rate (a) and 5-year DSF rate (b).

Begg’s and Egger’s tests indicated that there was no evidence of significant publication bias after assessing the funnel plot (Figures 4 and 5) for the studies included in our meta-analysis.

## Discussion

Information about the prognostic and predictive value of CXCR4 in breast cancer is limited. To our knowledge, this present meta-analysis is the first study to systematically evaluate the association between chemokine receptor CXCR4 and clinicopathological features and prognostic factors in breast cancer. In our study, a combined analysis of 13 clinical studies, which detected the CXCR4 antigen in whole tissue sections, revealed a poor prognostic outcome in patients expressing high levels of CXCR4. The results indicate that CXCR4 expression is significantly associated with lymph node status, distant metastasis and 5-year OS and DFS rates. When compared with nuclear expression, the expression of CXCR4 in the membrane and cytoplasm could be more significant for prognosis.

What makes CXCR4 account for the poor prognosis in breast cancer? On the one hand, CXCR4 is most commonly found in malignant cells from different types of cancer. At least 23 different cancers, including breast cancer, express this receptor [23]. Interestingly, Anja *et al*. reported that CXCR4 was present at a low level or absent in normal breast tissue but was highly expressed both in primary and metastatic breast tumors [24]. Hypoxia, vascular endothelial growth factor (VEGF), transcription factor nuclear factor-κB (NF-κB) and estrogen have been shown to upregulate CXCR4 expression in the tumor microenvironment, leading to cancer cell proliferation, resistance to apoptosis and local invasion [25,26]. In our study, we also found that CXCR4 expression was linked to lymph node status and distant metastasis. Previous studies have shown that CXCR4 and its chemokine ligand 12 (CXCL12) are two key factors in breast cancer metastasis [27]. CXCR4 signaling in response to CXCL12 mediates actin polymerization and pseudopodia formation and subsequently induces chemotactic and invasive responses [28]. Hence, CXCR4 and CXCL12 form an important signaling axis between tumor cells and the tumor microenvironment, with the interaction influencing the adhesion, migration and invasion of tumor cells, reflecting the strong association of CXCR4 with breast cancer metastasis. However, other studies have indicated that CXCR4 is highly expressed on cancer stem cells, and its activation maintains a stem cell population in tamoxifen-resistant breast cancer [29]. Cancer stem cells (CSCs) exhibit stem cell-like characteristics and gain the ability to regenerate the bulk of tumor cells while maintaining their self-renewing potential [30]. Hence, CXCR4^+^ cells could contribute to the development of therapeutic resistance and relapse in breast cancer. Furthermore, the CXCR4/CXCL12 axis could mediate the chemotaxis of cancer stem cells [31] that are involved in the metastasis of breast cancer stem cells and cancer cell survival and proliferation [32]. Therefore, CXCR4 could be a marker for poor prognosis and metastasis of breast cancer.

Some limitations exist in the present meta-analysis. First, the number of included studies was relatively small. Because these 3865 patients received different treatments (neoadjuvant therapy or just surgical resection), we were unable to assess the potential outliers present in individual studies. Second, all the studies were from North America or Asia. Distinct site differences are believed to exist and could cause publication bias. Finally, the applied method for detecting CXCR4 expression and the cutoff values were different in the included studies, which could cause heterogeneity among the studies. We could not perform subgroup analysis to explore this influence because few studies offered concrete data.

## Conclusions

In conclusion, despite the limitations listed above, the present study showed a significant correlation between CXCR4 expression and the 5-year OS and DFS rates in breast cancer patients. CXCR4 expression was also associated with lymph node status and distant metastasis. Thus, CXCR4 could have prognostic significance for patients with breast cancer. Recent studies indicated that small antagonists, such as AMD3100, inhibited the primary tumor and metastasis in animal models of breast cancer [33]. Hence, CXCR4 antagonists could have a significant therapeutic impact on primary and metastatic breast cancer by disrupting tumor vasculature in the microenvironment [34]. Furthermore, inhibiting CXCR4 in tumor cells has the potential to induce growth arrest or apoptosis and to prevent invasion and metastasis [34]. Hence, in addition to being a prognostic marker, CXCR4 could also be an anti-cancer therapy target. Recent preclinical studies in mouse models of leukemia have provided proof of concept for the greater benefits of combining CXCR4 inhibition with conventional chemotherapy relative to chemotherapy treatment alone [31,32]. We infer that in the case of breast cancer, a CXCR4 inhibitor could improve survival and prognosis.

Further studies are required to clarify the role of CXCR4 in predicting organ specificity in breast cancer metastasis; to explain whether CXCR4 could change in the process of breast cancer neoadjuvant therapy and whether this change could be significant for treatment and prognosis; and to evaluate the application of CXCR4 as a bio-marker for breast cancer prognosis and target therapy. Furthermore, anti-CXCR4 inhibitors need to be carefully assessed for possible side effects that could occur during immune cell trafficking or other biological processes.

## Competing interests

The authors declare that they no competing interests.

## Authors’ contributions

ZZ and CN participated in the design of the study and performed the statistical analysis. WC, PW, ZW and JY carried out the data extraction. FQ and HJ conceived of the study, and participated in its design and coordination and helped to draft the manuscript. All authors read and approved the final manuscript.

## Pre-publication history

The pre-publication history for this paper can be accessed here:

http://www.biomedcentral.com/1471-2407/14/49/prepub

## Supplementary Material

Additional file 1: Table S1 Relationship between CXCR4 expression and the clinicopathological features of breast cancer. Click here for file

Additional file 2: Table S2 Relationship between CXCR4 expression and the prognosis of breast cancer. Click here for file
