# Supplementary material for: Expression of CXCR4 and breast cancer prognosis: a systematic review and meta-analysis
Source: BMC Cancer. 2014 Jan 29;14:49. doi: 10.1186/1471-2407-14-49 (PMC3911796; doi:10.1186/1471-2407-14-49)
Supplement: Additional file 1: Table S1 — Relationship between CXCR4 expression and the clinicopathological features of breast cancer. [file 1471-2407-14-49-S1.doc]

**Table S1.** Relationship between CXCR4 expression and Clinical pathological Features of breast cancer

|  |  | T category  (T3,4 vs. T0~2) |  | N category  (Pos vs. Neg) |  | Distant metastasis  (M1 vs. M0) |  | ER  (Pos vs. Neg) |  | PR  (Pos vs. Neg) |  | CerbB-2  (Pos vs. Neg) |
| --- | --- | --- | --- | --- | --- | --- | --- | --- | --- | --- | --- | --- |
|  | N1^a^ | RR(95%CI) | N2 | RR(95%CI) | N3 | RR(95%CI) | N4 | RR(95%CI) | N5 | RR(95%CI) | N6^a^ | RR(95%CI) |
| Over all | 8 | 1.09(0.80-1.47) | 15 | 1.20(1.01-1.43) | 6 | 1.52(1.17-1.98) | 14 | 0.97(0.97-1.05) | 13 | 0.99(0.87-1.12) | 13 | 1.16(0.86-1.56) |
| Ethnicity |  |  |  |  |  |  |  |  |  |  |  |  |
| Caucasian | 4 | 1.11(0.70-1.78) | 6 | 0.98(0.80-1.19) | 2 | 1.76(0.91-3.42) | 5 | 0.91(0.81-1.04) | 4 | 0.90(0.68-1.19) | 4 | 0.83(0.48-1.42) |
| Asian | 4 | 1.07(0.72-1.58) | 9 | 1.40(1.15-1.72) | 4 | 1.39(0.65-2.99) | 9 | 1.04(0.93-1.16) | 9 | 1.04(0.92-1.17) | 9 | 1.37(1.04-1.81) |
| Staining pattern |  |  |  |  |  |  |  |  |  |  |  |  |
| Membrane/cytoplasm | 6 | 1.14(0.80-1.63) | 10 | 1.29(0.99-1.69) | 4 | 1.72(1.29-2.29) | 9 | 0.88(0.84-0.93) | 8 | 0.91(0.76-1.09) | 8 | 1.10(0.74-1.63) |
| Nuclear | 2 | 0.97(0.55-1.72) | 5 | 1.06(0.96-1.17) | 2 | 0.91(0.48-1.73) | 5 | 1.02(0.97-1.08) | 5 | 1.09(0.97-1.23) | 5 | 1.28(0.79-2.07) |
| Follow time (month) ^b^ |  |  |  |  |  |  |  |  |  |  |  |  |
| <60 | 4 | 1.11(0.70-1.78) | 3 | 1.07(0.88-1.30) | 1 | 2.80(1.21-6.51) | 2 | 0.82(0.51-1.33) | 2 | 0.86(0.51-1.43) | 1 | 1.37(0.85-2.22) |
| ≥60 | 4 | 1.07(0.72-1.58) | 7 | 1.10(0.86-1.40) | 5 | 1.45(1.11-1.91) | 7 | 0.97(0.87-1.09) | 6 | 1.03(0.85-1.25) | 7 | 1.11(0.63-1.94) |
| Sample size ^c^ |  |  |  |  |  |  |  |  |  |  |  |  |
| <113 | 4 | 1.68(1.02-2.76) | 5 | 1.30(0.98-1.74) | 2 | 0.90(0.30-2.67) | 4 | 1.01(0.85-1.21) | 4 | 0.95(0.77-1.17) | 4 | 1.12(0.90-1.39) |
| ≥113 | 4 | 0.85(0.58-1.25) | 10 | 1.16(0.94-1.43) | 4 | 1.74(1.04-2.91) | 10 | 0.96(0.87-1.06) | 9 | 0.99(0.84-1.17) | 9 | 1.19(0.77-1.81) |

a: categories with zero frequency were excluded

b: median of follow-up time among all studies

c: median of sample size among all studies
