# Supplementary material for: Expression of CXCR4 and breast cancer prognosis: a systematic review and meta-analysis
Source: BMC Cancer. 2014 Jan 29;14:49. doi: 10.1186/1471-2407-14-49 (PMC3911796; doi:10.1186/1471-2407-14-49)
Supplement: Additional file 2: Table S2 — Relationship between CXCR4 expression and the prognosis of breast cancer. [file 1471-2407-14-49-S2.doc]

**Table S2.** Relationship between CXCR4 expression and prognosis of breast cancer

|  |  | OS rate  (survive vs. dead) |  | DFS rate  (survive vs. dead) |
| --- | --- | --- | --- | --- |
|  | N1 | RR(95%CI) | N2 | RR(95%CI) |
| Over all | 7 | 0.77(0.70-0.86) | 6 | 0.70(0.59-0.83) |
| Ethnicity |  |  |  |  |
| Caucasian | 5 | 0.73(0.61-0.86) | 6 | 0.70(0.59-0.83) |
| Asian | 2 | 0.81(.072-0.91) | 0 | - |
| Staining pattern |  |  |  |  |
| Membrane/cytoplasm | 6 | 0.78(0.69-0.88) | 6 | 0.70(0.59-0.83) |
| Nuclear | 1 | 0.77(0.65-0.92) | 0 | - |
| Follow time (month) ^a^ |  |  |  |  |
| <60 | 5 | 0.73(0.61-0.86) | 6 | 0.70(0.59-0.83) |
| ≥60 | 2 | 0.81(0.72-0.91) | 0 | - |
| Sample size ^b^ |  |  |  |  |
| <113 | 7 | 0.77(0.70-0.86) | 6 | 0.70(0.59-0.83) |
| ≥113 | 0 | - | 0 | - |

a: median of followup time among all studies

b: median of sample size among all studies
